# Navigating change: a comparative analysis of health technology assessment reforms across agencies – processes, drivers, and interdependencies

**DOI:** 10.1017/S0266462325000133

**Published:** 2025-03-14

**Authors:** Gayathri Kumar, Priscila Radu, Patricia Cubi-Molla, Martina Garau, Eleanor Bell, Jia Pan, Ramiro Gilardino, Julie Van Bavel, Agnes Brandtmüller, Katherine Nelson, Melinda Goodall

**Affiliations:** 1 OHE – Office of Health Economics, London, UK; 2 UKHSA, London, UK; 3 Adelphi Values, London, UK; 4 MSD, Zurich, Switzerland; 5 MSD, Budapest, Hungary; 6 Drexel University, Philadelphia, PA, USA; 7 Goodall HTA Consulting Limited, Manchester UK

**Keywords:** technology assessment, biomedical, health care evaluation mechanisms, policy, methods

## Abstract

**Objectives:**

Health technology assessment (HTA) is a critical part of healthcare decision making in many countries. Changes in Methods and Processes (M&P) of HTA agencies can affect the time and degree of patient access to treatments. Published literature focuses on the different M&P adopted by HTA agencies, rather than on how these have come about over time. Our study investigates key HTA reforms and explores their drivers and interdependencies in a set of HTA agencies in Europe, Asia-Pacific, and North America.

**Methods:**

We conducted a targeted literature review on M&P guidelines and subsequent changes to those, for 14 HTA agencies. We supplemented and validated initial findings with 29 semi-structured interviews with country-specific experts. We used analytical tools to create process maps, proactivity and influence networks, and clusters of HTA agencies.

**Results:**

We found that processes leading to M&P reforms follow similar steps across HTA agencies. The three most important drivers to reforms were HTA practice and guidelines in other countries; the healthcare policy, legal, and political context within the agency’s country; and experience of challenges in the assessment by the HTA body itself. International collaborations have the potential to accelerate the evolution of HTA systems and the implementation of reforms.

**Conclusion:**

We identified PBAC (Australia), CDA-AMC (Canada), NICE (England), IQWiG (Germany), and ZIN (the Netherlands) as HTA agencies that are catalysts of HTA reforms as well as internationally influential. International collaborations may represent a useful route to accelerate changes as long as they ensure wide stakeholder engagement at an early stage.

## Introduction

Health technology assessment (HTA) is a critical part of healthcare decision-making in many countries. Current HTA agencies have different methods (their preferred technical approaches and practices on how to conduct HTA) and processes (steps followed and stakeholders involved in carrying out HTA). HTA methods and processes (M&P) can significantly impact recommendations made by HTA agencies (1) and have wide-ranging effects on patients, providers, industry, and society as a whole. HTA M&P can also influence patient access to new treatments and impact research and development (R&D) investment decisions. Therefore, HTA M&P should evolve in response to scientific advances, changes in societal preferences, methodological developments, and challenging political contexts.

Published literature compares different agencies’ M&P in a static way (international comparison of HTA M&P at a particular point in time (2;3), and generally exploring a single topic of interest (4–6)). Cross-border dynamics of HTA M&P (how guidelines evolve over time) are less analyzed in the literature (7), and usually focus on the emergence of HTA organizations, publication of their first guidelines (8–10), or refer to a specific topic (11). This article is the first attempt, to our knowledge, to document past full or partial HTA reforms, analyze drivers and processes leading to these reforms, and show how HTA agencies influenced each other in the development and reviews of their M&P guidelines. Understanding what lies behind HTA reforms is important for stakeholders to identify opportunities for engagement, inform evidence generation matching forthcoming HTA requirements, and support policy discussions.

This article aims to identify and analyze recent changes in HTA M&P, explore the processes and drivers for these changes, and analyze the dynamics between countries in terms of proactivity in implementing changes and the degree of influence between them. We considered a sample of HTA agencies in Europe, Asia-Pacific, and North America, chosen as a representative model for the breadth of approaches to HTA implementation.

## Methods

We conducted a targeted literature review and analyzed documents published from 2010 to 2023 related to M&P guidelines as well as changes made to those guidelines. Our research focused on HTA programs for pharmaceuticals, including medicines and vaccines, which start when a product is selected for assessment and concludes with a recommendation on funding within the healthcare system. Other types of health technologies (e.g., devices, digital therapeutics) and other activities that may be carried out by HTA agencies, including horizon scanning, were not included in the scope. We supplemented our findings with semi-structured interviews with country-specific HTA experts.

We investigated HTA agencies in 14 countries, as described in Box 1. These countries were chosen as examples of more established HTA agencies in Europe and the Asia-Pacific region.

Box 1. Full and abbreviated name of Health Technology Agencies for each country included in the study

**Table:** 

Acronym	HTA agency – full name	Country
PBAC	Pharmaceutical Benefits Advisory Committee	Australia
KCE	Belgian Health Care Knowledge	Belgium
CDA-AMC	Canadian Agency for Drugs and Technologies in Health	Canada
DMC	Danish Medicines Council	Denmark
NICE	National Institute for Health and Care Excellence	England
HAS	Haute Autorité de Santé	France
IQWiG	Institut fur Qualitat und Wirtschaftlichkeit im Gesundheitswesen	Germany
AIFA	Agenzia Italiana del Farmaco	Italy
INFARMED	National Authority of Medicines and Health Products	Portugal
ACE	Agency for Care Effectiveness	Singapore
AEMPS	Agencia Española de Medicamentos y Productos Sanitarios	Spain
TLV	Dental and Pharmaceutical Benefits Agency	Sweden
CDE	Centre for Drug Evaluation	Taiwan
ZIN	National Health Care Institute	The Netherlands

The pragmatic search of HTA agency websites and bibliographic databases was conducted in two stages. The first stage identified relevant documents published by the HTA agencies of interest and secondary literature relating to major changes in HTA M&P in general. In the second stage, we identified information specific to changes in the following topics: discount rates, modifiers, patient involvement in HTA (PI), real-world evidence (RWE), and surrogate end points. These topics were deemed particularly dominant in the recent HTA debate, both historically and with the aim of assessing innovative medicines. Data were extracted on: the timing of key M&P changes; qualitative descriptions of the policy changes and the agency’s positions on topics; drivers of reform; and references to other HTA agencies in the guidelines. Further details on the search strategy and data extraction protocol are shown in Supplementary Material 1. Our findings relate to changes in HTA M&P in general and specifically to the five HTA topics of interest.

Subsequently, we interviewed 29 experts with HTA experience with the agencies of interest (two experts per agency and an additional expert from the EUnetHTA collaboration). The interviews aimed to validate the literature review findings and elicit additional insights into the local context, including the interviewees’ views on the proactivity and influence of HTA agencies and opportunities and barriers to reforms. The interview guide is available in Supplementary Material 2.

We combined the findings from the literature review and interviews using several analytical tools. First, we tabulated the timings of country-specific HTA M&P updates, distinguishing between full and partial revisions. Second, we created a diagram to represent the process followed by each HTA agency to consider, discuss, and implement changes in the methods guidelines. Third, we created a framework that lists all the drivers that may trigger a review of the M&P or lead to the implementation of changes. The framework was based on the results from the literature review and was validated through expert interviews. Fourth, we provide the frequency that each driver in the framework was identified as an influence for changes in country-specific HTA M&P guidelines. Fifth, we created a network diagram representing the level of influence exerted by HTA agencies proxied by the number of times their M&P is referenced in other HTA agencies’ guidelines or related publications. We also created a heatmap of HTA agency proactivity, which presents the relative order in which countries implemented their first reform by topic. Finally, we grouped HTA agencies regarding their proactiveness to changes in M&P and the influence of those changes over other HTA agencies.

Dynamics between countries were identified by way of exploring (a) historical correlation that may occur because of the timeline; (b) historical causation (i.e., M&P changes by an HTA agency that are directly influenced by changes of another agency; and (c) prospective collaboration or agreements between countries to align on M&P and share learning.

## Results

We selected 374 publications in the literature search. Supplementary Material 3 presents the publication years of guidelines and updates identified in the review. We differentiate between full revisions of HTA guidelines and partial updates (if changes are only sought for specific sections in the guidelines). Before 2000, the agencies PBAC, CDA-AMC (previously CADTH), INFARMED, and ZIN had already published their HTA M&P guidelines – with full revisions in the case of PBAC and CDA-AMC (12–16). By 2016, all the countries in our list had their M&P guidelines published (17–28). We also observe that the revisions of these guidelines become more frequent over time.

### Process followed for HTA M&P reviews

Evidence on the reform process followed by PBAC, NICE, IQWiG, CDA-AMC, and DMC was found primarily in the literature, and it was more formally defined compared to the reform process followed by the other HTA agencies in scope, where interviewees’ input was key to retrieve it. As a result, there are varying opportunities and risks for stakeholder interactions throughout each agency’s process.

**Figure 1.**
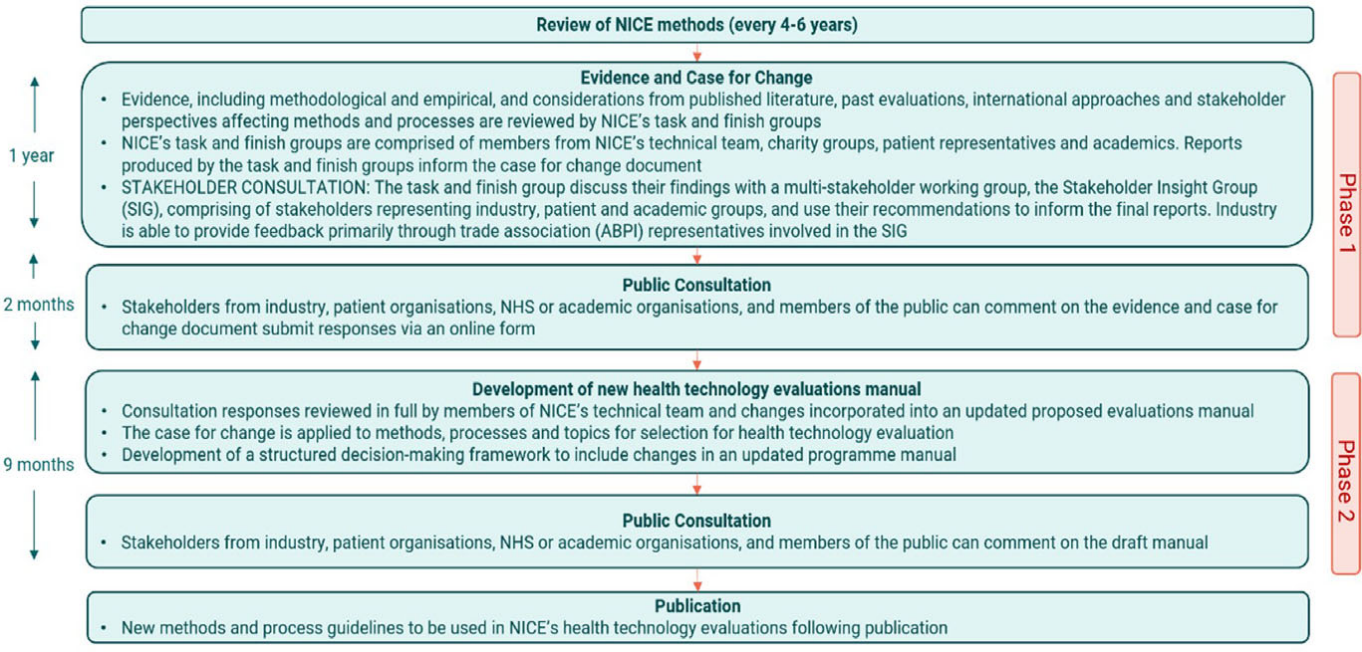
Process to guidelines and methods review in NICE. *Note that NICE process for M&P updates has changed to a modular approach where large reviews will no longer occur. We use this example because of its robustness and its relevance to past reviews, which are the focus of our analysis.

Our findings suggest that M&P reforms follow similar steps across HTA agencies, as depicted in the process map that reflects the process for NICE M&P updates (see Figure 1) – although timelines and the extent of stakeholder involvement may differ (29–32). The process illustrated in Figure 1 most applies to changes in the methods, as changes related to the processes often occur separately.

The reform process is usually initiated by a review of existing methods, with emphasis on identifying evidence supporting the case for change. The next stage includes a draft “proposal of change” document informed by the review and prior informal discussions with stakeholders to gather feedback and raise issues with the previous method updates. The “proposal of change” document may be accompanied by stakeholder meetings for some agencies to discuss findings and proposed changes (e.g., INFARMED and ZIN). A public consultation usually follows in which stakeholders from industry, patient organizations, academia, and members of the public are invited to share their views on the project. The format of the stakeholder consultation may be via online questionnaires or in-person interviews, in addition to several informal feedback points throughout the process. Feedback is incorporated into the final HTA methods guideline, which is subsequently published.

Major HTA M&P updates tend to happen in a 4- to 6-year cycle; nevertheless, when the need for a method update arises, these may be initiated outside of the update cycle. For example, NICE’s single technology appraisal process was introduced off-cycle in 2006 and was motivated by industry demand; and future NICE methods updates will use a modular and iterative approach when needed, to be more agile in reviewing and introducing updates in the future (33).

### Framework of HTA M&P drivers

We identified the drivers of M&P reforms and categorized these into three themes: stakeholders, country-specific context, and cross-border context. Stakeholders include HTA agencies, academics, patient representatives, healthcare professionals, industry, and society. The country-specific context refers to healthcare policy, legal, and political context. For the cross-border context, we highlighted the following drivers: scientific advances in health technologies and/or change in the R&D process; regulatory approval process and pathway changes; HTA practice or guidelines in other countries; and external shocks. Table 1 shows the complete list of drivers of changes in HTA M&P, their descriptions, and some examples identified in the literature review.

**Table 1.** List of drivers of changes in HTA M&P, description, and examples

Driver	Description	Example source
*Stakeholders*		
HTA agency	Experience or practical challenges in assessment Regular review process	“This revision… has involved substantial changes in many areas of the document. These changes have built on experience gained since the first revision of the guidelines was published in 1995 based on the experience of making decisions relying on cost-effectiveness.” (55) Guidelines “are usually reviewed annually with regard to any necessary revisions, unless errors in the document or relevant developments necessitate prior updating.” (56)
Academics	Publications on HTA M&P or methodological development by academic groups	“The revision process was driven by an external consultancy incorporating Australian and international experts reporting to a Guidelines Revision Steering Committee. It has benefited from extensive discussions among members of the Pharmaceutical Benefits Advisory Committee and its subcommittees, as well as a wide range of contributors from industry, government, academia, and the community.” (17)
Patient representatives	Patient association positions	“[…] it is generally considered important for HTA decisions to be made with patients’ awareness. They suggested that the main drivers for this change are patient representatives.”
Healthcare professionals	Healthcare professional association positions	“In contrast, academic stakeholders, NHS organizations and NICE committee members were more supportive.” (17;33)
Industry	Industry trade association and individual companies’ positions	“On 6 September 2021, the Commonwealth entered into a new Strategic Agreement in relation to reimbursement, health technology assessment and other matters (Strategic Agreement) with Medicines Australia acting on behalf of the innovator medicines industry. Under clause 5.3 of the Strategic Agreement, it was agreed that the Commonwealth would support and resource a Health Technology Assessment Policy and Methods Review.” (57)
Society	Societal value judgments on healthcare provision, societal preference studies	“There is evidence that society values highly health benefits in severe diseases, and it is legitimate that NICE values benefits in line with this societal value” (33)
*Country-specific context*		
Healthcare policy, legal, and political context	Change in HTA agency’s legal responsibilities	“Within the framework of the Act on the Reform of the Market for Medicinal Products at the beginning of 2011, the Institute’s responsibilities were extended to the assessment of the benefit of drugs with new active ingredients shortly after market entry… For this purpose, manufacturers must submit dossiers summarizing the results of studies…The new regulations in Section 35a SGB V are the basis for these assessments.” (22)
	Politician or policymaker willingness to address a health policy concern or set new policy objectives	“[The selection of criteria that appraisal committees take into account [has been an evolving process, partly informed by the deliberative process of the NICE Citizen’s Council and partly reflecting higher level concerns of the Department of Health and secretary of state.” (58)
*Cross-border context*		
Scientific advances in health technologies and/or change in the R&D process	Introduction of new types of health technologies	“[T]he change in the conditions underpinning the emergence of innovations reinforced the need to further increase the practice and quality of economic evaluation.” (34)
	Changes in global medicine development process	“This revision… reflects changes in the medicine development process internationally.” (17)
Regulatory approval process and pathway changes	Accelerate approvals	Feedback from an expert interviewee
HTA practice or guidelines in other countries	Emergence of guidelines overseas that drives best practice	“This was based on the NICE modifier, which corresponds to HTA practice or guidelines in other countries in our framework” (27)
External shocks	COVID–19, global economic crisis, inflation	Feedback from an expert interviewee

### Frequency of drivers by country

The frequency of mentions of drivers, both in the literature and predominantly by interviewees, in relation to a specific country is presented in Table 2. The three most important drivers are HTA practice or guidelines in other countries (18 instances across all countries); the healthcare policy, legal, and political context (16); and the HTA body itself (15). International best practices are considered by most of the HTA agencies explored in this study, except for AIFA and TLV, where evidence of such practices was not found. Updates to guidelines can also be triggered by a change in national government and subsequently policy, particularly for countries in which HTA M&P are closely intertwined with legal statutes. For example, we identified that interest rates in the country impacted the discount rate recommended by HAS and DMC (34;35).

**Table 2.** Frequency of drivers, as mentioned in the literature and by interviewees, of M&P reform relating to key HTA topics, by country

HTA: Health technology assessment; R&D: Research and development															
Driver	PBAC	KCE	CDA-AMC	DMC	NICE	HAS	IQWiG	AIFA	INFARMED	ACE	AEMPS	TLV	CDE	ZIN	Total
**Stakeholders**															
HTA body	2	1	3		3		1	1	1		2			1	**15**
Academics	1		1		2				2						**6**
Patients			2		1	1								1	**5**
Healthcare professionals		1			1										**2**
Industry	1		1		1				2						**5**
Society	1	1			5										**7**
**Country-specific context**															
Healthcare policy, legal, and political context		1	2	3	5			2	1				2		**16**
**Cross-border context**															
Scientific advances in health technologies and/or changes in the R&D process				1	1										**2**
Regulatory approval process and pathway changes															**0**
HTA practice or guidelines in other countries	1	2	1	1	3	1	1		3	1	1		1	2	**18**
External shocks															**0**
**Total**	**6**	**6**	**10**	**5**	**22**	**2**	**2**	**3**	**9**	**1**	**3**	**0**	**3**	**4**	

Stakeholders that drive M&P reforms include primarily the HTA body itself, followed jointly by patients and industry, then academics, and society and healthcare professionals. Many drivers stem from the need to improve on existing M&P for HTA, due to challenges with assessment throughout the process as well as arising from the HTA body’s internal perception on the topics of interest. Academic position, generally sought directly by the HTA agency, and updates to the methodology surrounding the topic were also shown to drive changes to HTA M&P. In general, HTA agencies seek to understand stakeholders’ needs and opinions, and evidence-based arguments from stakeholder groups can be a key driver to change.

Scientific advances in certain health technologies or changes in R&D processes may simply necessitate updated guidelines to assess the relevant intervention accurately; however, this driver was only explicitly mentioned twice across all countries. We did not find evidence that external shocks, such as the COVID-19 pandemic, and the regulatory approval process are a cause of changes for the countries in scope.

### Influence exerted by other HTA agencies

**Figure 2.**
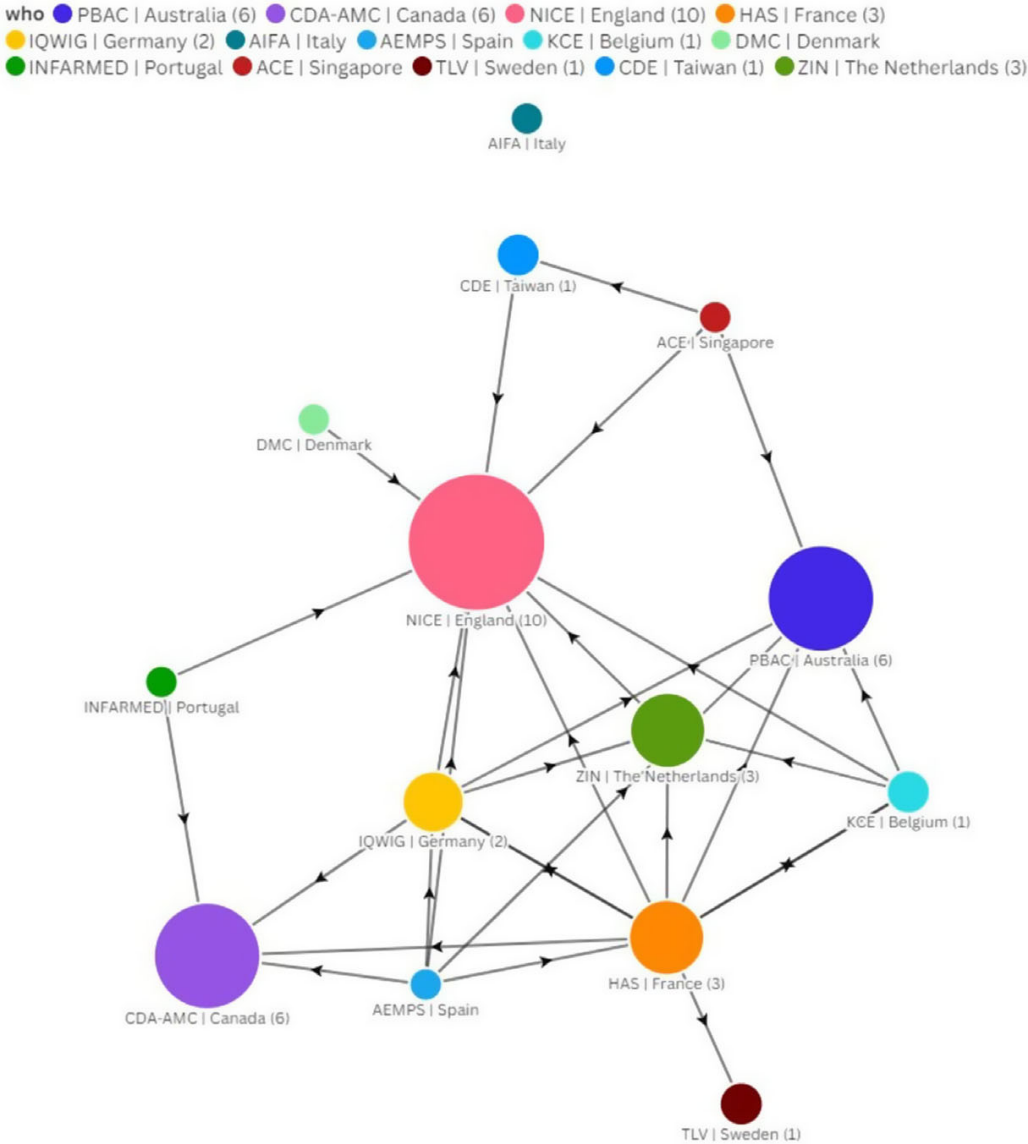
Network diagram of HTA agency influence. The direction of influence is represented by the direction of the arrowhead. For example, an arrow pointing from HAS to PBAC would mean that HAS mentions PBAC in its guidelines. A double-headed arrow indicates that both HTA agencies mention each other in their guidelines (e.g., CDA-AMC and PBAC). The number in brackets represents the number of times an agency is mentioned by other agencies included in the study, and the size of the nodes is proportional to that. Agencies that have no number in brackets are not mentioned by another agency (e.g., INFARMED). Likewise, agencies that neither mention nor are mentioned by another agency have no links (e.g., AIFA).

The number of times an HTA agency’s M&P is referenced in another HTA agency’s guidelines or publications is used as a proxy for their level of influence. Figure 2 depicts the direction of influence in a network diagram. PBAC, CDA-AMC, and NICE were referenced as the most impactful, while AIFA, AEMPS, DMC, INFARMED, and ACE were not referred to at all by other HTA bodies in scope.

### Heatmap of proactivity

**Figure 3.**
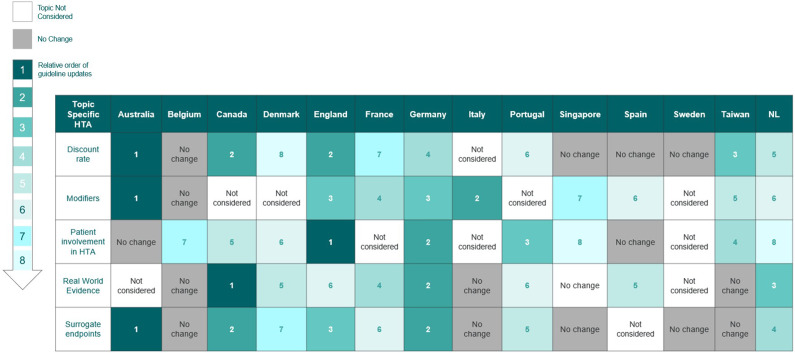
Heatmap of changes to HTA methods or processes. The heatmap depicts the relative ordering of M&P guidelines updates relating to each topic for the HTA agencies in scope. Gray cells indicate that there has been no change in the HTA agency’s stance on the topic since 2010. Non-shaded cells denote that the HTA agency does not explicitly refer to the topic in their guidelines. Health technology assessment; NL: The Netherlands.

Figure 3 highlights which HTA agencies were first to implement changes to their guidelines for each topic. The numbering indicates the relative position of their updates compared to the other countries. In cases where HTA agencies changed their guidelines in the same year, they were assigned the same relative position. Agencies that do not consider a topic in their guidelines or have not changed their guidelines since 2010 are represented by white and gray cells, respectively.

The HTA agencies that were first, second, or third movers in most topics were PBAC (first mover in discount rates, modifiers, and surrogate end points); NICE and IQWiG (among the top three movers in four topics); and CDA-AMC (three topics). AIFA, INFARMED, CDE, and ZIN were each second or third movers in one topic but were later (fourth onward) to move across other topics, to have no change, or not consider a topic at all in their guidelines. KCE, DMC, ACE, and AEMPS were fifth or later to move, if at all; and TLV either had no change or did not consider any of the topics.

The heatmap does not convey the level of innovation in an agency toward a specific topic, suggest comparability, or express the value judgment of different agencies or countries. Some countries may have had well-established evaluation methods for some topics from the outset and, therefore, did not require any changes, such as PBAC and TLV’s stated positions on accepting surrogate outcomes in the absence of final outcomes since 1995 and 2003, respectively. The stance is comparable with current guidelines from other agencies that may have enacted multiple or only fairly recent changes to reach the same position, such as INFARMED.

### Clusters of HTA agencies by proactivity and influence

We grouped HTA agencies into three clusters based on our analysis of proactivity and influence reported in previous sections, alongside insights provided by expert interviewees: *catalysts* (NICE, PBAC, ZIN, CDA-AMC, and IQWiG); *traditionalists* (HAS, TLV, and KCE); and *observers* (DMC, AIFA, INFARMED, ACE, AEMPS, and CDE).

### Catalysts

HTA agencies defined as *catalyst* are proactive in implementing M&P changes, and those changes impact other HTA bodies. Our findings highlight that NICE is the most proactive HTA agency, with more than four full revisions of its initial M&P guidelines. NICE is also identified as the most influential among the included agencies, with 10 other HTA agencies (CDA-AMC (36), HAS (37), IQWiG (38), AEMPS (39), KCE (18), DMC (35), INFARMED (40), ACE (41), CDE (27), and ZIN (28)) referencing NICE in their guidelines. Besides that, NICE International provides advisory services for international health organizations, ministries, and government agencies (42) and is involved in an international collaboration spanning three continents (43). NICE has also previously been actively involved in EUnetHTA Joint Actions (44).

Similar to NICE, PBAC stands out as a high-influence HTA body in our analysis. PBAC is mentioned in the HTA guidelines of six other agencies (KCE (18), CDA-AMC (19), HAS (37), IQWiG (45), AEMPS (39), and ACE (41)). Our findings also identify PBAC as a first mover in providing M&P guidelines updates related to discount rates, modifiers, and surrogate end points.

While ZIN implemented reforms in all topics, these changes were introduced at a relatively late stage – compared to the order of the agencies in our sample. ZIN has influenced the M&P guidelines of three other agencies (HAS (34), IQWiG (38), and KCE (46)). While not explicitly referenced in NICE’s 2022 M&P guidelines, ZIN’s proportional shortfall approach to capturing severity as a modifier has most likely influenced NICE’s approach to accounting for disease severity (47). ZIN also shows involvement in multiple international collaborations, such as the EUnetHTA Joint Clinical Assessment (JCA) Committee, EUnetHTA21, and EU IVDR (48–50).

CDA-AMC is also highly proactive in updating M&P guidelines around discount rates, RWE, and surrogate end points. CDA-AMC is referenced in the HTA guidelines of six other agencies (PBAC (13), HAS (51), IQWiG (38), AEMPS (39), INFARMED (40), and ACE (41)) and is involved in an international collaboration with eight other global HTA agencies (43). Our results also identify IQWiG as a relatively proactive and influential HTA agency, although to a lesser extent than the other *catalyst* agencies. HAS (34) and AEMPS (39) reference IQWiG are described in their guidelines.

### Traditionalists

We label HTA agencies as *traditionalist* if they exert some degree of influence over other HTA agencies and take a reactive approach to implementing changes in their M&P guidelines. HAS published its first M&P guidelines in 2011, and we only identified one full revision dating 2020. Topic-specific reforms were also relatively late within our sample, suggesting that HAS is generally reactive to HTA reforms. We consider HAS influential, as it is referenced in the guidelines of KCE (18), IQWiG (38) and AEMPS (39), and it contributes to international initiatives through its involvement in EUnetHTA. HAS’s early access process is often referred by other agencies, such as AIFA.

We observed the limited proactivity of TLV and KCE in instigating reform. Since the publication of the first M&P guidelines (TLV in 2003 and KCE in 2008), they were only reviewed once (partial review for TVL in 2017 and full review for KCE in 2012). TLV and KCE are considered moderately influential, as they are both referenced in HAS’ guidelines (37) and engaged in international collaborations, such as EUnetHTA and JNHB (Joint Nordin HTA-Bodies) (in the case of TLV) (52).

### Observers

We consider an HTA agency to be an *observer* if they are generally a “late mover” in implementing reforms and have little influence on other agencies’ M&P guidelines. For example, INFARMED was one of the first European HTA agencies to formalize the HTA M&P in a written document (15). However, it has been a “late mover” in reforms to HTA topics (exception for patient involvement in HTA), and updated M&P guidelines only in 2019, being less influential among the agencies in scope.

DMC, AIFA, and AEMPS have been “late movers” in implementing topic-specific reforms, and their M&P guidelines are not referenced by other agencies. DMC has shown signs of gradual involvement in the international debate via EUnetHTA, as well as its recent entry into the JNHB collaboration, while AIFA and AEMPS are engaged in the EUnetHTA collaboration.

ACE and CDE are also “late-movers” on M&P reforms. Although ACE references CDE’s guideline, no other agencies in this study have referred to ACE’s or CDE’s guidelines.

## Discussion

Our research identified variations among agencies in how formal and structured their M&P reform processes are. NICE is an example where there is a process with clear steps, including stakeholder involvement and opportunities for their input. Other agencies have less transparent or well-defined processes, which might make it challenging for external parties to anticipate, get involved, and contribute. This could represent a key priority for HTA agencies to address to ensure inclusivity and broad endorsement among local stakeholders.

Most of the drivers identified in the literature for change referred to the perspectives of different stakeholders, such as academics, patients, and HTA experts. We add that the foundation of evidence-based reforms should also include recent robust empirical evidence (including societal preference studies) and method development.

International collaborations have the potential to accelerate the evolution of HTA systems and the implementation of reforms by enabling agencies to anticipate and address common challenges in a timely and efficient manner. We observe an increase in international collaborations between HTA agencies. A recent collaboration includes eight agencies across Australia, Canada, and the United Kingdom (43), which aims to improve work sharing and collaborate on horizon scanning and methods development. Given that most agencies in this collaboration were classed as *catalysts* in our analysis, the authors expect that they will provide international leadership and be a crucial drive for HTA method evolution. EUnetHTA was one of the first examples of joint HTA work and information sharing among a large number of countries, and some of its principles have informed the method guidelines for the JCA, part of the Regulation of HTA in the European Union. We anticipate that the latter will have a predominant role in shaping the M&P of HTA systems in European member states in the coming years. Finally, Joint Nordic HTA Bodies (53) (previously known as FINOSE) provides an example of how neighbor countries with similar HTA systems can benefit from cooperation to promote the convergence of methods and efficient assessments. Going forward, collaborations should promote more streamlined and regular updates, similar to the modular approach that NICE is implementing, and also pool resources together to conduct initial literature reviews of HTA practices, identify emerging innovative methods, select those suitable for HTA practice, and pilot them jointly.

Finally, to be fully successful, collaborations should ensure full and early involvement of stakeholders, to increase the legitimacy of changes, improve evidence generation, and facilitate implementation of reforms at the national level.

As a limitation, our literature review only included publications in English, which might have led to the exclusion of relevant documents in local languages. Where identified as relevant by experts, additional non-English documents were added and machine-translated (Google Translate). Only a few documents related to CDE were professionally translated into English, as machine translation was not deemed appropriate. Language bias could have also impacted the reference to guidelines across HTA agencies, resulting in primarily English-speaking agencies (NICE, PBAC, and CDA-AMC) being more likely to be referenced by other agencies, and hence being considered as *catalysts.*

We have tried to mitigate this by validating the literature results with interviews with experts from all countries. An additional way to further limit this could be to include experts who speak the language of each country considered in the writing process. Furthermore, we encourage similar research to occur in other regions where the same languages are spoken, such as Spanish-speaking communities in South America.

We also note that, as our study focuses on more established agencies in Europe and south-east Asia, there is a risk that our choice of HTA agencies may not be representative of other areas where HTA is in development or nascent. The exclusion of emerging HTA agencies could also influence the generalizability of the conclusions.

The core literature review was conducted from January 2000 to April 2022. Additional updates published between the end dates of the searches and September 2023 were identified on an ad hoc basis. We also note that PBAC was surrounded by a policy and methods review at the time this article was written; therefore, its current reform processes and drivers might not be reflected here.

Evidence on the drivers was not extensive. However, it is important to note that documents related to past reforms are often removed from agency websites, and specific factors leading to individual reforms may only appear in agencies’ committee or board papers that were not included here. We did not make assumptions on potential interactions between drivers, only reporting on how they were mentioned within the literature and by expert interviews.

The interview process was based on a limited sample size, meaning that some experiences or views on past reforms may not have been captured adequately. We also restricted the number of interviewees to two experts per HTA agency. This impacts the analysis of those HTA agencies with less detailed or specific M&P guidelines, allowing more room for flexibility in practice. In those cases, our findings from the literature review do not entirely align with the experts’ opinions. For example, interviewees noted that TLV focuses on changing the application of methodology in practice rather than changing the documented guidelines; and this may explain the observed limited proactivity of the TLV in instigating reforms.

Finally, it is important to note that our results depict influence and proactivity in relative rather than absolute terms. Although the list of countries in scope is extensive, the relative positions can change with the addition (or exclusion) of other HTA agencies to the scope. For example, the interviews revealed that several countries in Latin America and Asia are developing their M&P based on CDA-AMC guidelines; that PBAC’s M&P guidelines serve as a model for the HTA approach in Japan; and the influence of INFARMED among the HTA agencies of Greece, Romania, and Cyprus. However, those links were out of this project’s scope and are not reflected in our clustering exercise.

Further research should explore the impact of HTA reforms on a set of quantitative metrics, including timelines to recommendations, degree of patient access to interventions, and patient outcomes; and qualitative ones, including quality of stakeholders’ submissions and of the decision-making process. Specifically, in the context of EU HTA regulation, new research can map its impact on national HTA M&P guidelines after a few years of implementation. Collaborations across agencies and, more generally, the research community should define and test optimal processes for M&P updates and their implementation. An example is provided by the framework developed by Jiu et al. (54) for the introduction of novel HTA methods.

## Conclusion

To the best of the author’s knowledge, this article is the first attempt to document past full or partial HTA reforms and analyze the drivers and processes leading to these reforms, including how HTA agencies have influenced each other in the development and reviews of their M&P guidelines. They identified PBAC, CDA-AMC, NICE, IQWiG, and ZIN as HTA agencies that are catalysts of HTA reforms as well as internationally prominent. NICE, PBAC, and CDA-AMC are among the agencies with the most influence on the M&P guidance of other countries. International collaborations (such as the recent one between HTA agencies in Australia, Canada, and the United Kingdom, as well as the Nordic collaboration) represent a valuable route to accelerate changes and ensure comprehensive stakeholder engagement at an early stage. These alliances could create convergence between HTA guidelines and provide international leadership in methods change. This could be beneficial for those agencies with limited or no guidance on certain topics. However, their success depends on how the national legislative framework and political objectives are addressed.

Future research should assess how HTA reforms impact HTA systems, such as timelines to develop recommendations, degree of patient access to interventions, and, in the longer term, patient outcomes.

## Supporting information

Kumar et al. supplementary material Kumar et al. supplementary material

## Supplementary material

The supplementary material for this article can be found at http://doi.org/10.1017/S0266462325000133.
