# Supplementary material for: Navigating change: a comparative analysis of health technology assessment reforms across agencies – processes, drivers, and interdependencies
Source: Int J Technol Assess Health Care. 2025 Mar 14;41(1):e21. doi: 10.1017/S0266462325000133 (PMC12018853; doi:10.1017/S0266462325000133)
Supplement: Kumar et al. supplementary material [file S0266462325000133sup001.zip › Supplementary material 1.docx]

# Supplementary material 1

**Search strategies** and main results

**Table 1** Search strategy (first search)

| Dimension/Item | Search command | Search [#] |
| --- | --- | --- |
| Population | (Europ* OR Canada OR Canadian OR Australia OR Australian OR England OR English OR British OR United Kingdom OR France OR French OR Germany or German OR Spain OR Spanish OR Italy OR Italian) .ti,ab. | 75 |
| Intervention | AND  (((health technology assessment) OR pharmacoeconomic) adj2 (guideline* OR manual* OR guidebook)).ti,ab. |  |
| Key content | n/a * |  |

#: number of documents retrieved, removing duplicates; *: no search command has been added

**Figure 1** PRISMA diagram of results from first search


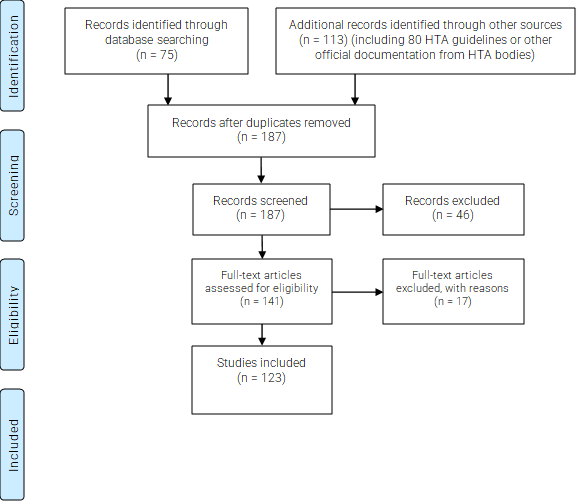


**Table 2** Search strategy (second search)

| Dimension/Item | Search command | Search [#] |
| --- | --- | --- |
| Population | (Europ* OR Canada OR Canadian OR Australia OR Australian OR England OR English OR British OR United Kingdom OR France OR French OR Germany or German OR Spain OR Spanish OR Italy OR Italian) .ti,ab | 559 |
| Intervention | AND  ((health technology OR pharmacoeconomic) adj2 (assessment OR institution* OR agenc* OR agency OR agencies OR expert* OR organi*ation* OR institute* OR guideline OR manual OR decree* OR policy OR regulation* OR rule*)) .ti,ab  AND ((evolution OR changes OR new OR update* OR novel OR innovat* OR latest OR recent)).ti,ab |  |
| Key content |  |  |
| General | n/a * | (66) |
| RWE | AND  (Real-world evidence OR RWE).ti,ab |  |
| Surrogate endpoints | AND  (surrogate* OR novel*).ti,ab AND (endpoint*).ti,ab |  |
| Severity, end of life, innovation and rare disease modifiers | AND  (modifier* OR severe OR severity OR end of life OR end-of-life OR innov* OR rare OR rarity OR orphan OR highly specialized OR highly specialised).ti,ab |  |
| Discounting | AND  (discount*).ti,ab |  |
| Assessment of additional indications | AND  (additional indication* OR multi-indication)ti,ab |  |
| Patient involvement in HTA | AND  ((patient*) adj2(expert* OR representativ* OR group* OR input* OR involvement)).ti,ab |  |
| HTA remit | AND  (remit* OR scope* OR mandate*).ti,ab |  |
| Implementation of simplified HTA procedure | AND  (procedure* OR process* OR method*) adj2 (simplif* OR streamlin* OR condens* OR consolidat* OR integrat*).ti,ab |  |

**Figure 2** PRISMA diagram of results from second search


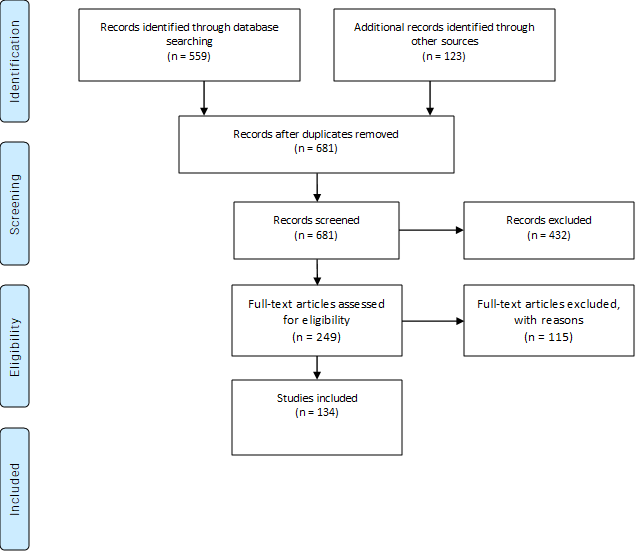


**Table 3** Country specific guidelines and complementary sources

| **Most relevant literature**  **(Official HTA guidelines)** | | |
| --- | --- | --- |
|  | **Official HTA guidelines** | **Other relevant documents from the website of the HTA agency** |
| Australia | (PBAC, 2016a)  (PBAC, 2013)  (PBAC, 2008)  (PBAC, 2006)  (PBAC, 2002)  (PBAC, 2000)  (PBAC, 1995) | (Australian Government Department of Health, 2021a)  (Australian Government Department of Health, 2021c)  (Australian Government Department of Health, 2021d)  (Australian Government Department of Health, 2021b)  (Australian Government Department of Health, 2019)  (Australian Government Department of Health, 2011)  (Australian Government Department of Health, 2009)  (PBAC, 2016b) |
| Canada | (CADTH, 2020)  (CADTH, 2018c)  (CADTH, 2006)  (CADTH, 1997) | (CADTH, 2021)  (CADTH, 2018b)  (CADTH, 2018a)  (CADTH, 2018d)  (CADTH, 2017a)  (CADTH, 2017b)  (PMPRB, 2021) |
| England | (NICE, 2022c)  (NICE, 2019)  (NICE, 2018a)  (NICE, 2018b)  (NICE, 2017a)  (NICE, 2017c)  (NICE, 2016a)  (NICE, 2014)  (NICE, 2013a)  (NICE, 2013b)  (NICE, 2011)  (NICE, 2009a)  (NICE, 2009b)  (NICE, 2009c)  (NICE, 2008a)  (NICE, 2006)  (NICE, 2004)  (NICE, 2001) | (NICE, 2022a)  (NICE, 2022b)  (NICE, 2022g)  (NICE, 2022h)  (NICE, 2021a)  (NICE, 2021b)  (NICE, 2020b)  (NICE, 2020c)  (NICE, 2016b)  (NICE, 2009a)  (NICE, 2008b)  (NICE DSU, 2022)  (NICE DSU, 2013)  (DHSC and NICE, 2018) |
| France | (HAS, 2022).  (HAS, 2020a)  (HAS, 2020b)  (HAS, 2012) | (HAS, 2019)  (HAS, 2014) |
| Germany | (IQWiG, 2022)  (IQWiG, 2020)  (IQWiG, 2017)  (IQWiG, 2015)  (IQWiG, 2013)  (IQWiG, 2011)  (IQWiG, 2009)  (IQWiG, 2008)  (IQWIG, 2006)  (IQWIG, 2005) | (Gemeinsamer Bundesausschuss, n.d.). |
| Italy | (AIFA, 2020)  (AIFA, 2018) |  |
| Spain | (AEMPS, 2013) | (RedETs, 2016)  (Puig-Junoy et al., 2014) |
| **Secondary literature** | | |
| (Allen et al., 2017)  (Angelis, Lange and Kanavos, 2018)  (Balijepalli C. et al., 2019)  (Bossi et al., 2020)  (Charlton, 2020)  (Dawoud et al., 2022)  (Earnshaw and Lewis, 2008)  (EUnetHTA, 2017)  (Favaretti et al., 2009)  (Fortinguerra et al., 2020)  (Fricke and Dauben, 2009)  (Goel, Mahajan and Chatterjee, 2020)  (Granados et al., 2000)  (Grigore B. et al., 2020)  (Hailey, 2009)  (Hofmann et al., 2021)  (Kim, Byrnes and Goodall, 2021)  (Kleijnen et al., 2016)  (Komakoma and Yi, 2022)  (Kristensen et al., 2019)  (López-Bastida et al., 2010)  (Menon and Stafinski, 2009)  (Miot and Thiede, 2017)  (Mostardt et al., 2014)  (Paris and Belloni, 2013)  (Ramsey et al., 2005)  (Ruof et al., 2014)  (Serrano-Aguilar et al., 2019)  (Skedgel, 2016)  (Sorenson, Drummond and Kanavos, 2008)  (Tarricone et al., 2021)  (Taylor and Weston, 2016)  (The Access Delivery Partnership, 2017)  (Toumi et al., 2017)  (Ubago Pérez et al., 2017)  (Wang et al., 2020)  (Ward et al., 2022)  (Zechmeister-Koss I., Schnell-Inderst P., and Zauner G., 2014)  (Zhang and Garau, 2020)  (Zhou et al., 2022)  (Zisis K., Naoum P., and Athanasakis K., 2021) | | |

**Table 4** Search strategy (first search)

| Dimension/Item | Search command | Search [#] |
| --- | --- | --- |
| Population | (Taiwan OR Taiwanese OR Singapore OR Singaporean OR Netherlands OR Dutch OR The Netherlands OR Belgium OR Belgian OR Flemish OR Denmark OR Danish OR Portugal OR Portuguese OR Sweden OR Swedish).ti,ab. | 36 |
| Intervention | AND  (((health technology assessment) OR pharmacoeconomic) adj2 (guideline* OR manual* OR guidebook)).ti,ab. |  |
| Key content | n/a * |  |

#: number of documents retrieved, removing duplicates; *: no search command has been added

**Figure 3** PRISMA diagram of results from first search


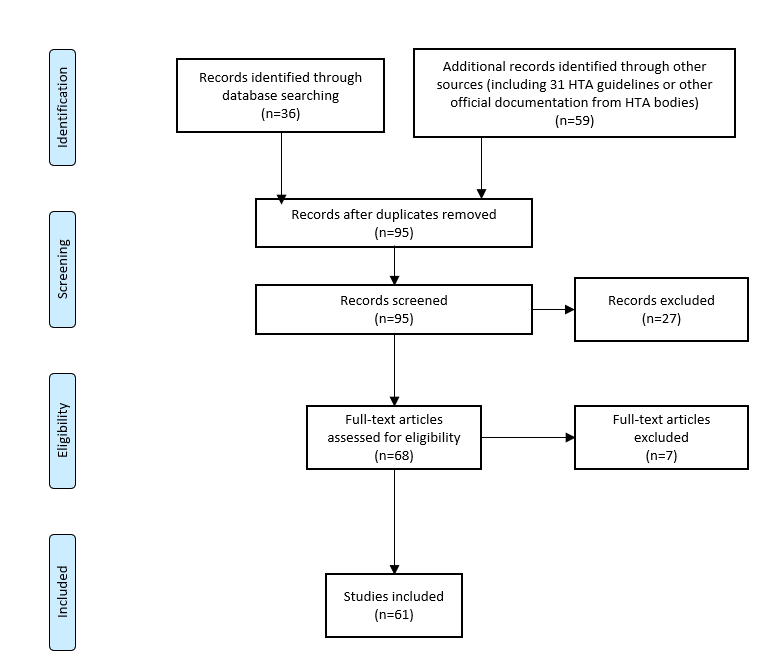


**Table 5** Search strategy (second search)

| Dimension/Item | Search command | Search [#] |
| --- | --- | --- |
| Population | (Taiwan or Taiwanese or Singapore or Singaporean or Netherlands or Dutch or The Netherlands or Belgium or Belgian or Flemish or Denmark or Danish or Portugal or Portuguese or Sweden or Swedish).ti,ab. | 259 |
| Intervention | AND  ((evolution or changes or new or update* or novel or innovat* or latest or recent) and ((health technology or pharmacoeconomic) adj2 (assessment or institution* or agenc* or body or bodies or expert* or organisation* or institute* or guideline or manual or decree* or policy)) and (guideline* or manual* or decree* or policy or regulation* or rule*)).ti,ab |  |
| Key content |  |  |
| General | n/a * | (56) |
| RWE | AND  (Real-world evidence OR RWE OR real world evidence OR real life data OR real life evidence OR real-world data OR real world data OR RWD).ti,ab |  |
| Surrogate endpoints | AND  (surrogate) AND (endpoint OR endpoints OR outcome OR outcomes).ti,ab |  |
| Severity, end of life, innovation and rare disease modifiers | AND  (modifier* OR severe OR severity OR end of life OR end-of-life OR innov* OR rare OR rarity OR orphan OR highly specialized OR highly specialised).ti,ab |  |
| Discounting | AND  (discount*).ti,ab |  |
| Assessment of additional indications | AND  (additional indication* OR multi-indication OR multi indication).ti,ab |  |
| Patient involvement in HTA | AND  ((patient*) adj2(expert* OR representativ* OR group* OR input* OR involvement)).ti,ab |  |
| HTA remit | AND  (remit* OR scope* OR mandate*).ti,ab |  |
| Implementation of simplified HTA procedure | AND  (procedure* OR process* OR method*) adj2 (simplif* OR streamlin* OR condens* OR consolidat* OR integrat*).ti,ab |  |

**Figure 4** PRISMA diagram of results from second search


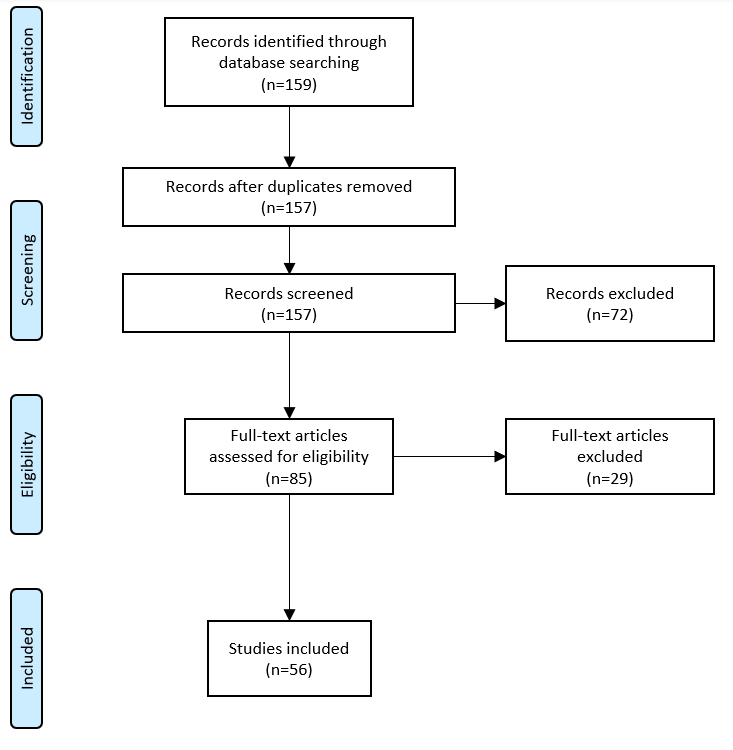


**Table 6** Country specific guidelines and complementary sources

| **Most relevant literature**  **(Official HTA Guidelines)** | | |
| --- | --- | --- |
| **Country** | **Official HTA guidelines** | **Other relevant documents from the website of the HTA agency** |
| Belgium | (Cleemput and Neyt, 2012)  (Cleemput et al., 2008) | (KCE, 2022b)  (KCE, 2019)  (KCE, 2021)  (KCE, 2022a) |
| Denmark | (DMC, 2021c, b; a) | (Om Medicinrådet, 2022)  (Danish Medicines Council, 2022) |
| Portugal | (INFARMED, 1998, 2019) | N/A |
| Singapore | (ACE, 2021)  (ACE, 2023d) | (ACE, 2022b, a, c; e; f) |
| Sweden | (TLV, 2003, 2017) | (TLV, 2019) |
| Taiwan | (TaSPOR, 2006)  (CDE, 2013) | (Centre for Drug Evaluation, Taiwan, 2022) |
| The Netherlands | (National Health Care Institute, 2016b, 2008) | (National Health Care Institute, 2018, 2016c; a) |
| **Secondary literature** | | |
| (Chen, Chang and Chang, 2018)  (Chen, Huang and Gau, 2022)  (Chiu, Pwu and Gau, 2015)  (Cleemput and Wilder, 2009)  (Duke NUS, ACE and CoRE, 2021)  (Enzing et al., 2021)  (EUnetHTA, 2013)  (Finansministeriet, 2021)  (Heintz et al., 2014)  (Hoomans T. et al., 2012)  (Kao et al., 2019)  (Lou et al., 2020)  (Makady A. et al., 2017)  (Ministerie van Volksgezondheid, 2015)  (NBHW, 2015)  (Pearce et al., 2019)  (Pereira et al., 2021)  (PPRI, 2017)  (Pwee, 2009)  (Reckers-Droog, van Exel and Brouwer, 2018)  (SBU, 2020a)  (Schurer et al., 2022)  (Segar et al., 2021)  (Shah et al., 2014)  (Simões and Augusto, 2017)  (Skedgel et al., 2022)  (Tan, 2021)  (Thiry et al., 2014)  (Vandijck and Annemans, 2010)  (WHO, 2015)  (Yang, 2017) | | |
