# Supplementary material for: Navigating change: a comparative analysis of health technology assessment reforms across agencies – processes, drivers, and interdependencies
Source: Int J Technol Assess Health Care. 2025 Mar 14;41(1):e21. doi: 10.1017/S0266462325000133 (PMC12018853; doi:10.1017/S0266462325000133)
Supplement: Kumar et al. supplementary material [file S0266462325000133sup001.zip › Supplementary material 2.docx]

# Supplementary material 2

**Interview guide:** Understanding the processes and drivers of HTA reforms

Interview guide and background

Introduction

PROJECT BACKGROUND

Health Technology Assessment (HTA) is a multidisciplinary process that evaluates the properties and effects of a health technology to support healthcare decision-making. Increasingly, HTA agencies conduct their assessments according to explicit and formal methodology that stipulates which evidence should be provided and analysed, and how it should inform decisions. Some HTA agencies also have formal processes, which define how the operational aspects of HTA – such as selecting technologies for assessment, timelines and milestones of the assessment, stakeholders’ involvement– should be conducted. HTA M&P are continually evolving, which poses challenges for manufacturers developing evidence in support of their submissions and for other stakeholders providing inputs to value assessments. HTA practices can have a significant impact on recommendations by HTA agencies and therefore affect patients, providers, industry, and society.

We are therefore undertaking a study aimed at understanding the triggers and sources that lead to reforms in M&P being adopted by HTA agencies. We define methods as the scientific approach used by HTA agencies to assess the effectiveness and cost effectiveness of health technologies, and the approach adopted by decision committee to develop and reach a recommendation based on the evidence assessment (appraisal). We define processes as the operational practices used to undertake HTA assessments and appraisals.

Our focus is on the core value assessment process, which starts when a technology is selected for assessment and concludes with a recommendation on provision or decision on funding within the healthcare system. Not included in our scope are other activities which may sometimes be carried out by HTA agencies, including horizon scanning and pricing. Our focus is on M&P for HTA of pharmaceuticals; therefore not in scope is HTA of other types of health technology such as vaccines, devices and diagnostics.

INTERVIEW OVERVIEW

We will be speaking to experts in HTA across a range of countries including Australia, Canada, England, France, Germany, Italy, Spain, Singapore, Taiwan and pan-European.

The aim of these interviews is to:

Validate and fill in any gaps of our initial mapping of HTA changes over time.

Understand the motivations for and constraints to HTA reforms, both prospectively and historically.

Discuss the appetite for change of individual agencies in relation to HTA M&P and to challenges debated in the research and policy community, both prospectively and historically.

The semi-structured interview will last one hour and will be carried out virtually at a time of your convenience by two members of the OHE team. Questions will focus on your experience and understanding of HTA in your country.

You will receive a brief pre-read document in advance to the interview. The document will be used to facilitate the discussion on the relevant topics.

DATA COLLECTION AND MANAGEMENT

The interview will be recorded for research purposes only. You will be asked whether you are happy for the recording to begin before the recording starts. As well as the interviewer, there will be another member of the OHE team on the line to take notes.

The identity of the interviewees, the data from the recording, and the notes will not be shared outside of the OHE project team and will be stored in a secure folder that only the OHE project team have access to. The sponsor of the research is blinded to the identity of the interviewees. The data (transcripts, recordings, notes taken by the notetaker) will not be shared with the sponsor of the research. When the project has been completed, the attributable recordings and the verbatim notes will be deleted. Only anonymised quotes will be used in the final report and will be identified only by country and area of expertise (i.e., specific job titles will not be used). Information provided by interviewees that could be used to identify their identity will not be included in the report.

Question outline

**Table 22** Questionnaire

| **#** | **Question** |
| --- | --- |
| **Section 1: Introductions and background (5 minutes)** | |
| **Section 2: HTA reform process (20 minutes)** | |
| 1 | Please refer to the draft process map in your pre-read.  - How far does this capture the process for M&P reviews at your country’s HTA agency?  - Would you make any change (in terms of indicated dates and method change)? If so, what are they?  - Please provide any references or sources where possible. (These can be provided by email after the interview - we will follow up with our requests). (Where there is uncertainty, date ranges may be useful.) |
| 2 | What are the key opportunities for industry throughout the HTA review process used at your country’s HTA agency (from engagement to implementation of change)? What are the risks? |
| **Section 3: Past and future HTA reforms (20 minutes)** | |
| 3 | - Do you agree with the key method changes of your country’s HTA agency that we indicate in our timelines slide of the pre-read?  Are we missing any important one? If so, what are they?  - Out of the changes discussed, what were the ones with the most significant impact on HTA decisions?  - Does the driver framework presented in slide 4 explain the changes discussed? Have we missed anything?    Can you think of one HTA reform that has been considered but not implemented? Were there any barriers to the introduction of this change? If yes, what were they? |
| 4 | In your pre-read, we have highlighted some key drivers to specific topics within HTA methods.  - Do you agree with the drivers we have pulled out from the literature?  There are some missing drivers for some topics that we have highlighted in red, are you able to suggest them? |
| 5 | Are there any key HTA reforms on the horizon for your country’s HTA agency?  - If so, what are the attitudes within the organisation to these reforms?  - Are there any other major challenges on the horizon for the agency? |
| Section 4: Culture relating to HTA reforms (5 minutes) | |
| 6 | How would you position your country’s HTA agency in relation to leadership in HTA methods development? Why? |
| 7 | How would you position your country’s HTA agency when it comes to initiating a new approach in HTA (HTA reforms)? Are you able to provide an example? |
| Section 5: HTA agencies influence (5 minutes) | |
| 8 | Which HTA agency/ies in your region and/or internationally do you perceive to be most proactive in implementing new approaches in HTA (HTA reforms)? |
| 9 | Which HTA agency/ies in your region and internationally do you perceive to be most influential for HTA reforms overall? |
| 10 | To what extent is your country’s HTA agency influenced by the HTA debate internationally?    To what extent is your country’s HTA agency influenced by changes in other countries?    Are HTA reforms by any specific HTA agencies particularly influential for your country’s HTA? |
| Section 6: Wrap up (5 minutes) | |
| 11 | Are there any other thoughts you wish to share on this topic today? |
| 12 | Thank you for your participation. We will now develop a summary note of our discussions today and share this with you for your confirmation that we have accurately represented your views. |
