# Supplementary material for: Navigating change: a comparative analysis of health technology assessment reforms across agencies – processes, drivers, and interdependencies
Source: Int J Technol Assess Health Care. 2025 Mar 14;41(1):e21. doi: 10.1017/S0266462325000133 (PMC12018853; doi:10.1017/S0266462325000133)
Supplement: Kumar et al. supplementary material [file S0266462325000133sup001.zip › Supplementary material 3.docx]

# Supplementary material 3

**Table 1** **Timeline of changes to M&P guidelines for pharmaceuticals**.

V1 = first version published; Full revision = substantive changes throughout methods and/or process guidelines, normally involving publication of a new version; Partial revision = changes to subsections of methods and/or process guidelines, normally involving publication of an updated version or addendum; Interim = guideline written by HTA agency, not published; Informal = guideline not written by HTA agency, published

| **HTA body** | **Type** | **pre-2000** | **2000** | **2001** | **2002** | **2003** | **2004** | **2005** | **2006** | **2007** | **2008** | **2009** | **2010** | **2011** | **2012** | **2013** | **2014** | **2015** | **2016** | **2017** | **2018** | **2019** | **2020** | **2021** | **2022** |
| --- | --- | --- | --- | --- | --- | --- | --- | --- | --- | --- | --- | --- | --- | --- | --- | --- | --- | --- | --- | --- | --- | --- | --- | --- | --- |
| **PBAC (Australia)** | M&P | V1 **(1990)**  Full (1992)  Full (1995) |  |  | Partial |  |  |  | Full  Partial | Partial | Partial |  |  |  |  | Partial |  | Partial | Full |  |  |  |  |  | [F] |
| **KCE (Belgium)** | M |  |  |  |  |  |  |  |  |  | V1 |  |  |  | Full |  |  |  |  |  |  |  |  |  |  |
| **CADTH (Canada)** | M | V1 **(1994)** Full **(1997)** |  |  |  |  |  |  | Full |  |  |  |  |  |  |  |  |  |  |  | Full |  |  |  |  |
| **DMC (Denmark)** | M&P |  |  |  |  |  |  |  |  |  |  |  |  |  |  |  |  |  | V1 | Partial | Partial | Partial | Partial (V1)  Full (V2) | Partial (V1)  Partial (V2) |  |
| **NICE (England)** | M | Interim **(1991)** |  | V1 |  |  | Full |  |  |  | Full |  |  |  |  | Full |  |  |  |  |  |  |  |  | Full |
|  | P |  |  | V1 |  |  | Full |  | Full |  |  | Full |  |  |  |  | Full |  |  |  | Full |  |  |  | Full |
|  | P (HST) |  |  |  |  |  |  |  |  |  |  |  |  |  |  | V1 |  |  |  | Full |  |  |  |  |  |
|  | P (CDF) |  |  |  |  |  |  |  |  |  |  |  |  |  |  |  |  |  | V1 |  | Full | Partial |  |  |  |
| **HAS (France)** | M |  |  |  |  |  |  |  |  |  |  |  |  | V1 |  |  |  |  |  |  |  |  | Full |  |  |
| **IQWiG (Germany)** | M&P |  |  |  |  |  |  |  |  |  |  | V1 |  |  |  |  |  | Full |  |  |  |  |  |  |  |
| **AIFA (Italy)** | M&P |  |  |  |  |  |  |  |  |  |  |  |  |  |  |  |  |  |  |  |  |  | V1 |  |  |
|  | TI |  |  |  |  |  |  |  |  | V1 |  |  |  |  |  |  |  |  |  | Full |  |  |  |  |  |
| **INFARMED (Portugal)** | M | V1 (1998) |  |  |  |  |  |  |  |  |  | Full |  |  |  |  |  |  |  |  |  |  |  |  |  |
| **ACE (Singapore)** | M&P |  |  |  |  |  |  |  |  |  | V1 | Partial |  | Partial |  |  |  |  |  |  |  |  |  |  |  |
| **AEMPS (Spain) + CatSalut (Catalonia)** | M&P |  |  |  |  |  |  |  |  |  |  |  | Informal (Method) |  |  |  | V1 Cat (Method) |  |  | V1 (Method) |  |  | Partial (Process) |  |  |
| **TLV (Sweden)** | M |  |  |  |  | V1 |  |  |  |  |  |  |  |  |  |  |  |  |  | Partial |  |  |  |  |  |
| **CDE (Taiwan)** | M |  |  |  |  |  |  |  | Informal |  |  |  |  |  |  | Full |  |  |  |  |  |  |  |  |  |
| **ZIN (The Netherlands)** | M&P | Guidelines for pharmacoeconomic research (1999) |  |  |  |  |  | Partial | Partial |  | Partial |  | Partial |  |  |  |  |  | Full |  |  |  |  |  |  |
