# Supplementary figures and images for: Navigating change: a comparative analysis of health technology assessment reforms across agencies – processes, drivers, and interdependencies
Source: Int J Technol Assess Health Care. 2025 Mar 14;41(1):e21. doi: 10.1017/S0266462325000133 (PMC12018853; doi:10.1017/S0266462325000133)

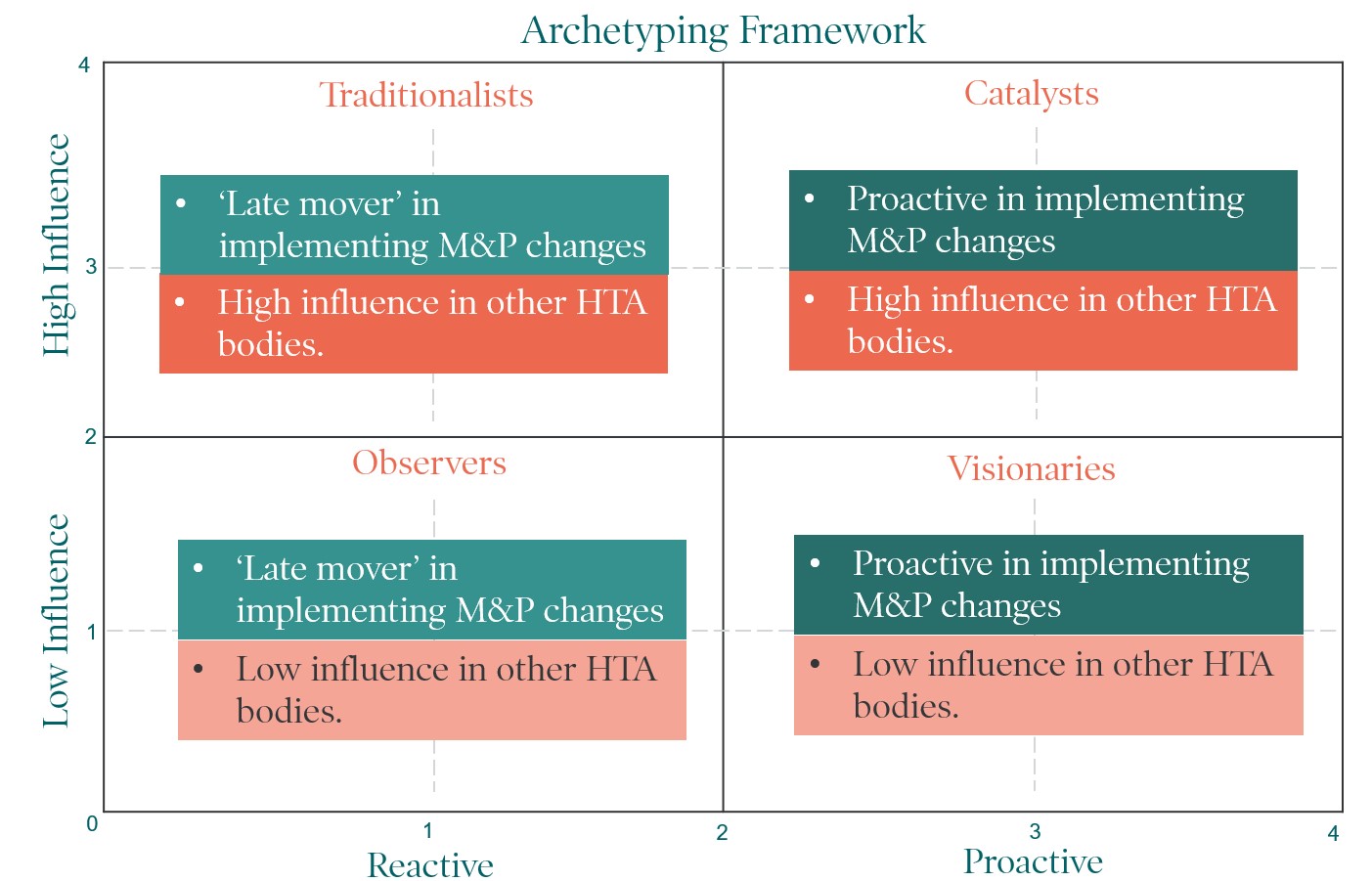

Supplement: Kumar et al. supplementary material [file S0266462325000133sup001.zip › Supplementary fig. HTA agencies grouped by proactivity and influence.jpg]
